# Bubble Melt Electrospinning for Production of Polymer Microfibers

**DOI:** 10.3390/polym10111246

**Published:** 2018-11-10

**Authors:** Ye-Ming Li, Xiao-Xiong Wang, Shu-Xin Yu, Ying-Tao Zhao, Xu Yan, Jie Zheng, Miao Yu, Shi-Ying Yan, Yun-Ze Long

**Affiliations:** 1 Collaborative Innovation Center for Nanomaterials & Devices, College of Physics, Qingdao University, Qingdao 266071, China; liyeming313@163.com (Y.-M.L.); wangxiaoxiong@qdu.edu.cn (X.-X.W.); yushuxin326@163.com (S.-X.Y.); ytzhao1995@163.com (Y.-T.Z.); my2373@columbia.edu (M.Y.); ysy5954418@163.com (S.-Y.Y.); 2 Industrial Research Institute of Nonwovens & Technical Textiles, College of Textiles & Clothing, Qingdao University, Qingdao 266071, China; yanxu-925@163.com (X.Y.); zhengjie2009123@126.com (J.Z.); 3 Department of Mechanical Engineering, Columbia University, New York, NY 10027, USA

**Keywords:** bubble melt electrospinning, polymer microfibers, polyurethane, polylactic acid

## Abstract

In this paper, we report an interesting bubble melt electrospinning (e-spinning) to produce polymer microfibers. Usually, melt e-spinning for fabricating ultrafine fibers needs “Taylor cone”, which is formed on the tip of the spinneret. The spinneret is also the bottleneck for mass production in melt e-spinning. In this work, a metal needle-free method was tried in the melt e-spinning process. The “Taylor cone” was formed on the surface of the broken polymer melt bubble, which was produced by an airflow. With the applied voltage ranging from 18 to 25 kV, the heating temperature was about 210–250 °C, and polyurethane (TPU) and polylactic acid (PLA) microfibers were successfully fabricated by this new melt e-spinning technique. During the melt e-spinning process, polymer melt jets ejected from the burst bubbles could be observed with a high-speed camera. Then, polymer microfibers could be obtained on the grounded collector. The fiber diameter ranged from 45 down to 5 μm. The results indicate that bubble melt e-spinning may be a promising method for needleless production in melt e-spinning.

## 1. Introduction

In the last 20 years, electrospinning (e-spinning) technology has become one of the main methods for producing ultrafine fibers [1,2], and this technology also has a rapid development both in fabricating functional fibers and potential applications. The electrospun (e-spun) fibers have been applied in various fields, such as biomedicine, tissue engineering, filtration, textiles, and nanodevices, because of their many excellent characteristics, such as large surface area to volume ratio, high porosity, and microscale to nanoscale diameter [3,4,5,6]. As is well known, the needle is easily blocked sometimes during solution e-spinning. Hence, many designs of needleless e-spinning have been proposed to solve the problem. For instance, a two-layer system, with a ferromagnetic suspension at the bottom and a polymer solution above it, can produce nanofibers [7]. A conical metal wire can be used for producing nanofibers, and cylinder and disk nozzles can also replace the needle [8]. He et al. reported a bubble solution e-spinning, where the output of fibers is higher than in traditional e-spinning. The diameter of fibers fabricated by this method is usually below 100 nm. He et al. also produced some different shapes, such as helix fibers and even nanoparticles [9,10,11,12,13]. Bubble solution e-spinning has its obvious advantages compared with traditional e-spinning, such as the spinning process with no blockage, the throughput is high and producing fiber membrane of high porosity [14,15]. When the e-spun fibers were used in biomedicine and tissue engineering, the shortcomings in traditional e-spinning, such as toxic solvent and low productivity, obviously restrict further applications in these fields [16,17,18,19]. Traditional solution e-spinning has its inherent defects which may hinder the way to solve these problems, particularly those of toxic solvent, solvent evaporation, and residual solvent [20,21,22]. Melt e-spinning, another branch of e-spinning, has attracted widespread attention in recent years. Many researchers have paid much attention to melt e-spinning, particularly the morphology adjustment and the potential application of microfibers. In 1981, Larrondo and Manley published the first paper on melt e-spinning. However, the second study on melt e-spinning was not published until 2001, by Reneker and Rangkupan [23,24]. Since the devices for melt e-spinning experiments are more complicated, and the fiber diameter is larger than the conventional solution e-spun fibers, the research work on melt e-spinning is relatively less than that on solution e-spinning, although melt e-spinning is a technique without toxic and solvent evaporation [25,26,27]. Melt e-spinning controls the fiber membrane more precisely than meltblown, but the needle is also frequently and easily blocked, which is the main shortcoming for high productivity [28,29,30,31]. In order to increase productivity, some progress has been made in the past several years, for example, Yang et al. used umbellate spinneret for mass production of ultrafine fibers [32]. Fang et al. produced polypropylene (PP) fibers by a rotary metal disc spinneret without needle block [33]. However, no research about bubble melt electrospinning has been reported. Detailed research on this topic would provide a reference for mass production using melt electrospinning.

Here, we propose a bubble melt e-spinning for fabricating polymer microfibers. Since the viscosity of polymer melt is much larger than polymer solution, this is a challenge for the bubble melt e-spinning. When compressed air was passed into polymer melt, many bubbles were formed on the surface of the polymer melt. When these bubbles were exposed to the electrostatic field between the polymer melt and the collector, the bubbles burst, and some jet flows were generated from the “Taylor cones” of the burst bubbles. Then, polymer microfibers were deposited on the collector. The effects of different parameters on the fiber morphologies were also investigated.

## 2. Experimental Section

### 2.1. Materials

Polylactic acid (PLA) with a molecular weight of ~20,000 and polyurethane (TPU) with a molecular weight of ~100,000 were purchased from Dongguan Thriving Plastic Raw Materials Co., Ltd. (Dongguan, China). All the chemicals were directly used without further processing.

### 2.2. Melt E-Spinning Setup

**Figure 1:**
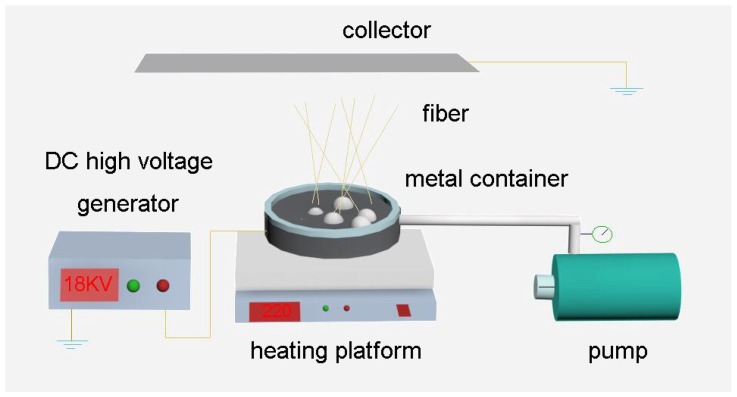
Schematic drawing of the bubble melt e-spinning setup.

The bubble melt e-spinning device was assembled by ourselves, which consisted of a gas pump, a DC high voltage generator (DW-P4-3-1ACCC, Tianjin, China), a thermostatic heating platform (200 × 200 mm^2^, 0–400 °C, 800 W, Shenzhen, China), a metal container (150 × 150 mm^2^) and an aluminum foil as collector. A metal conduit connected the air pump at one end, and the other end was fixed at the bottom of the metal container, which was placed on the thermostatic heating platform. The aluminum foil collector was over the top of the container, and the distance between collector and container could be adjusted. The positive and negative electrodes connected with the collector and the metal container, respectively. Figure 1 shows the schematic illustration of the bubble melt e-spinning setup.

### 2.3. E-Spinning Process

The polymer (TPU or PLA) particles were put into the metal container which was placed on the thermostatic heating platform. Then, turning on the heating platform, the heating process would last for several minutes until the polymer particles became a melt. The air pump was turned on gradually to create bubbles from the surface of polymer melt. Once the bubbles were generated on the surface of polymer melt, the DC high voltage generator was turned on to form an electrostatic field between the collector and the metal container. Through the high-speed camera, we could observe directly that multiple jets were ejected from the burst bubble towards the collector during the melt e-spinning process.

### 2.4. Characterization

The morphologies and structures of the melt e-spun polymer fibers were measured and characterized by a scanning electron microscope (SEM, Hitachi TM-1000, Tokyo, Japan) and a Fourier transform infrared (FTIR) spectrometer (Thermo Scientific Nicolet iN10, Shanghai, China). These samples were coated with an evaporated Au thin film before SEM characterization. A rheometer (MCR301, Anton Paar, Shanghai, China) was used to test the rheological properties of the polymer melt at different temperatures. The melt e-spinning process was recorded by a high-speed camera (Photron, UX50, Tokyo, Japan). X-ray diffraction (XRD) was tested using a Rigaku SmartLab X-ray diffractometer using Cu-Kα radiation (λ = 1.54178 Å) with an acceleration voltage of 40 kV. Thermogravimetric analysis (TGA) of the fiber membranes was performed from on a TGA2 (Mettler Toledo, Zurich, Switzerland), from 25 to 500 °C.

## 3. Results and Discussion

**Figure 2:**
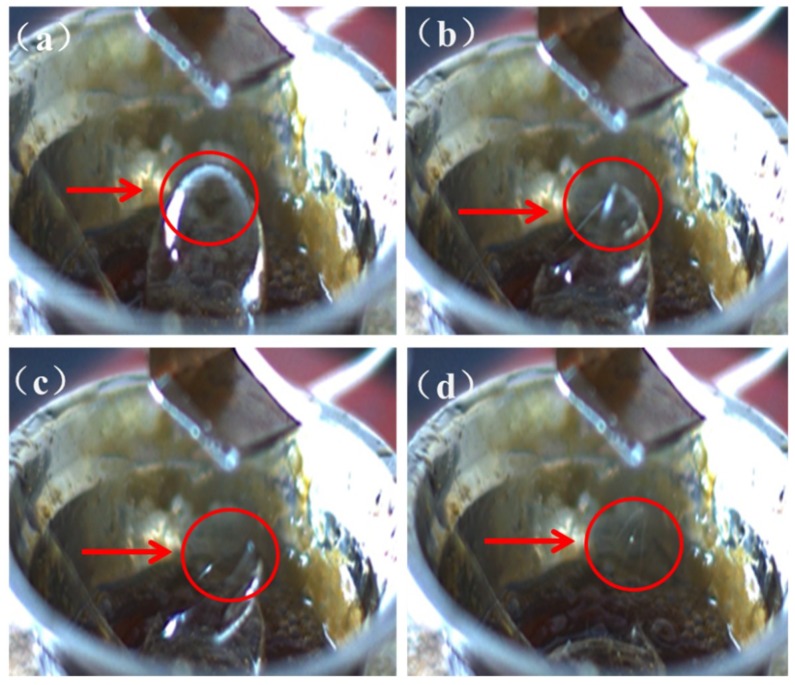
The photos of the unstable bubble and the burst bubble formed fibers: (**a**) the bubble formed; (**b**) the bubble burst; (**c**) the conical tip uplifted; and (**d**) the fibers formed.

When the bubble melt e-spinning started, several bubbles were generated on the polymer melt surface (please see the video in the Supplementary Materials). In order to see the e-spinning process more clearly, Figure 2 only shows a big bubble. In fact, there were many bubbles generated in the spinning process. The bubbles gradually became a cone while the applied voltage was close to the threshold value (Figure 2a). Then, increasing the gas pressure and the voltage to exceed the threshold value, the bubbles became unstable and then burst with multiple jets ejected toward the collector. In the process, several bubbles and conical tips which played the role of Taylor cone in the traditional e-spinning process were uplifted after the bubble burst (Figure 2b–d). The number of bubbles and jets mainly depends on the viscosity of polymer melts, gas pressure and the air flow rate, the applied voltage, and the distance between the surface and collector. These parameters can be adjusted in the e-spinning process to produce more bubbles and jets.

**Figure 3:**
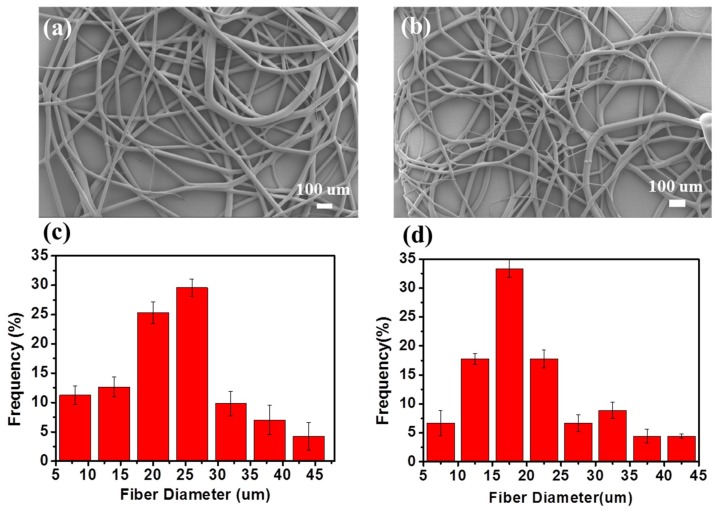
(**a**,**b**) SEM photograph and (**c**,**d**) diameter distribution of the melt e-spun polyurethane (TPU) fibers.

Figure 3a,b shows some typical SEM images of the melt e-spun TPU microfibers, taken at different positions of the collector. As shown in the picture, most of the fibers are disordered. Besides straight fibers, some helical fibers, thick fibers (~45 µm) and thin fibers (~5 µm) also can be observed. Figure 3c,d shows the diameter distribution maps. Most of the fibers have a diameter of about 20 µm. However, we can see from the pictures that a few fibers are thinner than others. According to the experiment, we analyzed that it was simply because the broken bubbles were different from each other both in size, viscosity, and distance to the collector. Multiple jets generated from bubbles with a small size and thinner bubble wall were more likely to form thinner fibers. In addition, the distance between the bubble tip and the collector was also different for bubbles with different sizes. Hence, it is still a challenge to produce stable uniform fibers. In this experiment, the difficulties are obvious compared to the common solution e-spinning, because the viscosity of the polymer melt is larger. At the same time, the bottom-up way for e-spinning needs to overcome the gravity. As a result, the diameter of the fabricated fibers by this method is larger. However, throughput of the fibers produced by bubble melt e-spinning is higher than the traditional melt e-spinning with a needle. Then, fibers fabricated by bubble are more scattered than the traditional one; at the same time, the porosity is higher.

**Figure 4:**
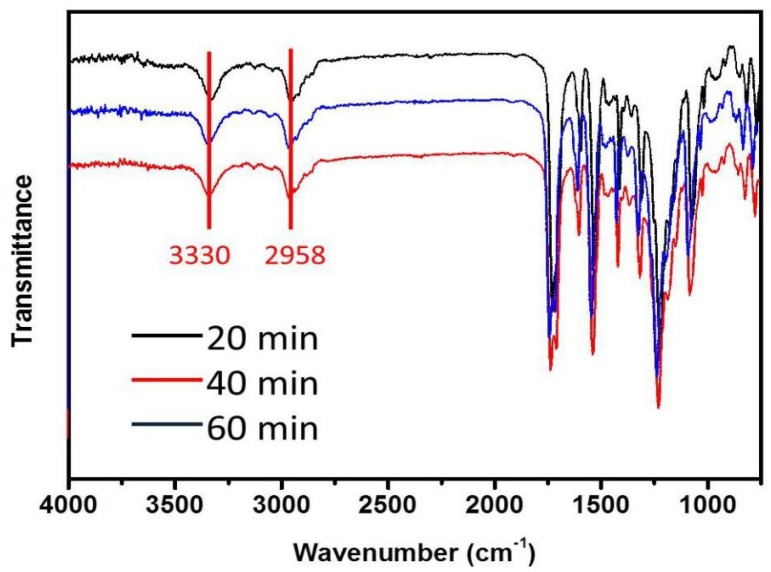
Infrared spectra of TPU with different heating times during the experiment.

Figure 4 shows the infrared spectra of the TPU fibers that were e-spun at different heating times during the experiment (heating temperature was 240 °C, heating time was 20, 40, and 60 min from the start of the experiment). From the infrared test results, we could also see that the TPU was stable in the melt e-spinning process. The peak at 3330 cm^−1^, which indicates the N–H group in urethane (–NHCOO–), and the peaks at 2958 cm^−1^, which belongs to the asymmetric and symmetric vibration of the –CH_2_ group, respectively, are the characteristic peaks of TPU [34,35]. However, in our experiment, the color of the TPU melt gradually changed into light yellow with increasing heating time, which may be the result of partial thermal degradation of the polymer [36,37].

**Figure 5:**
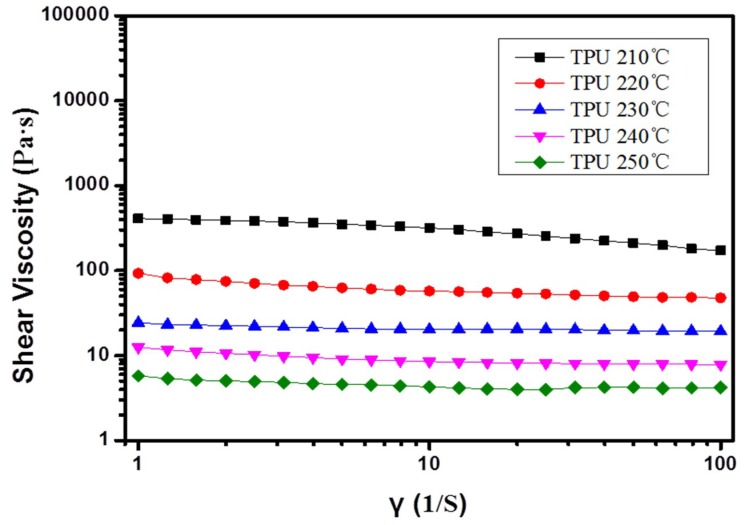
Rheological properties of TPU at different temperatures.

Figure 5 shows the rheological properties of the TPU at different temperatures. The results also exhibit the mechanism of this melt e-spinning experiment. At the same temperature, the viscosity curve is stable. The shear viscosity decreases obviously with the temperature rising at the same shear rate. Hence, at the beginning of the experiment, we need to heat the materials for a long time. Otherwise, the viscosity is too high to prevent the formation of the bubbles. Therefore, keeping the heating temperature at about 210 °C is necessary for the fibers’ formation. When the temperature is above 270 °C, the viscosity of the polymer melt is too low to obtain stable bubbles. We would see many jets directly formed from the surface of the polymer at the same time. However, these jets are not stable, and the resultant fibers are too thick. When these fibers are collected at the collector, it will prevent the normal process of the e-spinning. Keeping a proper temperature during the experiment is the key factor to the success of the experiment.

**Figure 6:**
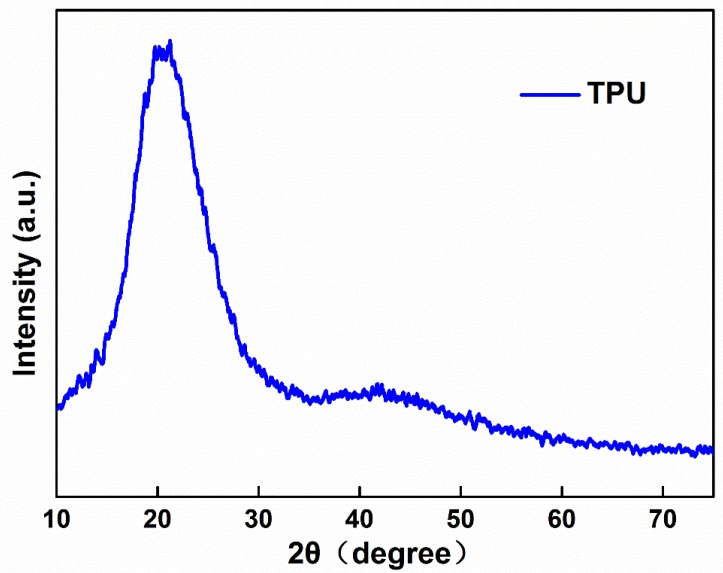
XRD Patterns of TPU fibers.

Figure 6 shows the XRD patterns of TPU fibers fabricated by bubble melt electrospinning. From the picture, we can see that the TPU had a clear crystalline peak after electrospinning. A broad amorphous band peaked at around 2θ = 20°. This indicates that the TPU remains stable after being spun into fibers. This result also proves that the prepared TPU fibrous membrane has low crystallinity.

**Figure 7:**
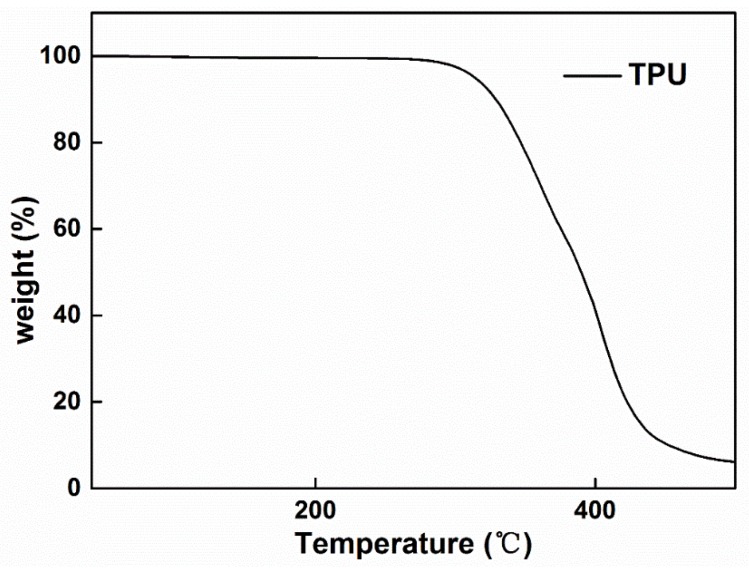
TGA patterns of TPU fibers.

Figure 7 shows the TGA test of the TPU fibers produced by bubble melt electrospinning. we can see that the TPU fibers stay stable before temperature reaches 300 °C. Our experiment was usually carried out between 200 and 300 °C, therefore, the properties of TPU are not changed.

**Figure 8:**
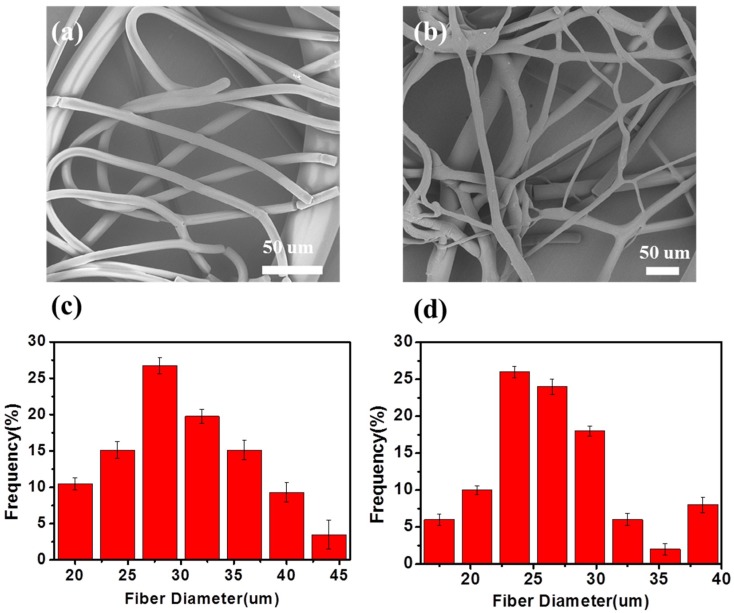
(**a**,**b**) SEM photograph and (**c**,**d**) diameter distribution of the melt e-spun PLA fibers.

PLA microfibers were also fabricated by this method, as shown in Figure 8a,b. The e-spun PLA microfibers are fragile, which can be clearly seen from the broken fibers in the picture [38]. Figure 8c,d shows the diameter distribution maps. The average fiber diameter is about 30 µm. Compared to the TPU fibers e-spun by this method, the average diameter of PLA fibers is larger. However, PLA microfibers were also successfully produced by the bubble melt e-spinning, indicating that it is an effective method to fabricate various polymer microfibers.

**Figure 9:**
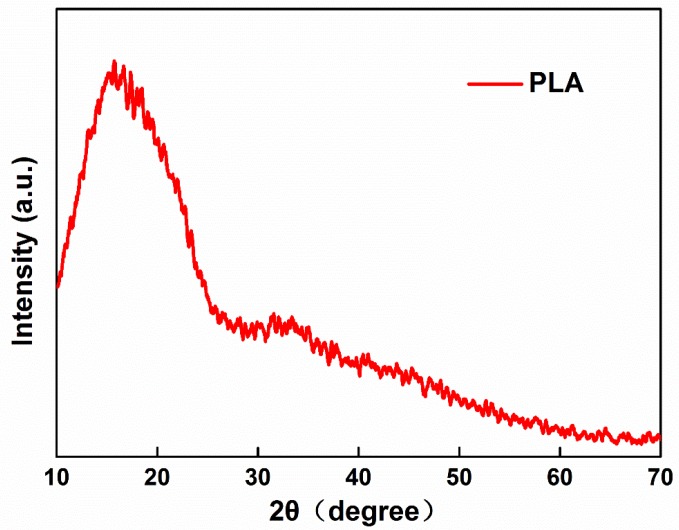
XRD Patterns of PLA fibers.

Figure 9 shows the XRD test of the PLA fibers produced by bubble melt electrospinning. The patterns show us that the electrospun PLA fibers had a clear crystalline peak. The broad amorphous band peaked at around 2θ = 17° and matches the common PLA XRD patterns. The result means that the properties of electrospun PLA fibers stay stable. The PLA did not change during the bubble melt e-spinning process.

**Figure 10:**
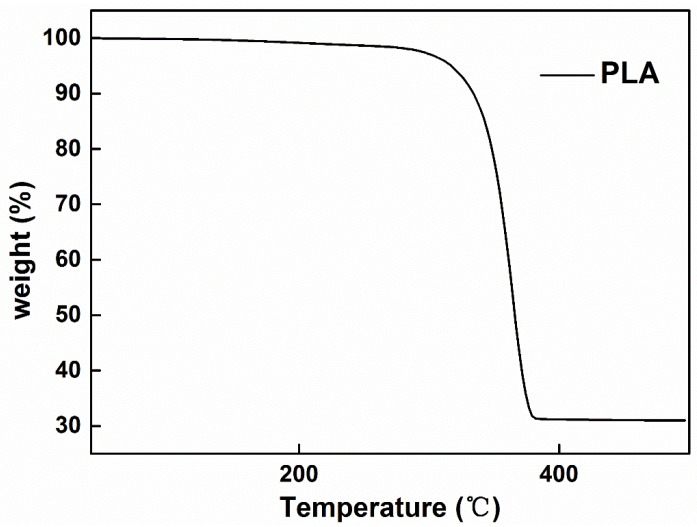
TGA patterns of PLA fibers.

Figure 10 shows the TGA test of the PLA fibers produced by bubble melt electrospinning. We can see that the PLA fibers stay stable before the temperature reaches 280 °C. Our experiment was usually carried out between 200 and 250 °C, indicating that our experiment can be carried out successfully.

**Figure 11:**
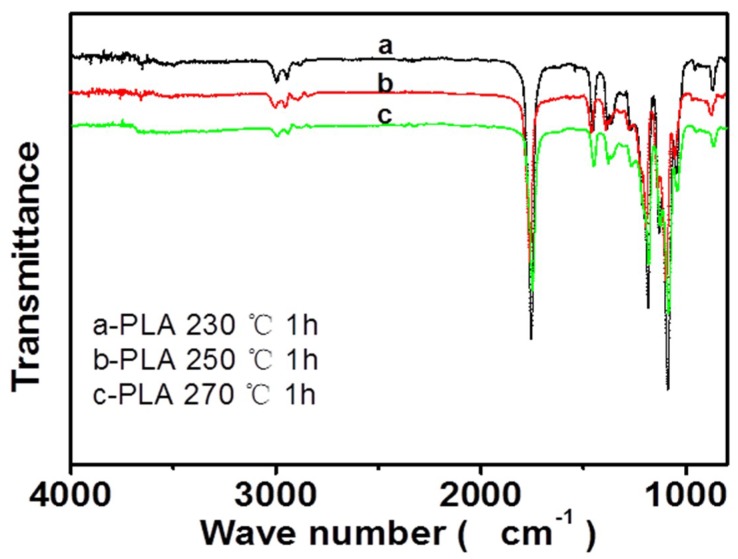
Infrared spectra of melt e-spun PLA fibers at different temperatures.

Figure 11 shows the infrared spectra of the PLA fibers that were melt e-spun at different temperatures (230, 250, and 270 °C) with the heating time of about one hour. The peak at 1758 cm^−1^ indicates the exist of a carbonyl group, the peak at 1132 cm^−1^ is assigned to the C–O symmetric telescopic vibration, and the bending vibration peak of CH_3_ is at 1455 cm^−1^. Here, it is noted that during our experiment, the color of the PLA melt also gradually changed into light yellow over time, just like the TPU melts. Hence, we want to know whether the color change of PLA melts has some relationship with heating temperature. The results in Figure 11 clearly show that the PLA melts remained stable at different temperatures (230, 250, and 270 °C). Namely, no obvious thermal degradation of PLA melts was observed even at 270 °C for a heating time of one hour.

## 4. Conclusions

A new method, bubble melt e-spinning, has been proposed in this paper, which is an effective way to fabricate fibers in melt e-spinning. It may be a promising needleless e-spinning method for mass production in melt e-spinning. The device of the experiment, as shown in our work, was easy to assemble and operate in certain conditions. Obviously, the bubble melt e-spinning method is an environmentally friendly and efficient technique. It overcomes some defects, like needle congestion, low yield, and toxic and mutual interference in the common solution e-spinning process. This e-spinning system may be applied for mass production in melt e-spinning. The quality of fibers and the experimental device will be developed in further research.

## Supplementary Materials

The following are available online at http://www.mdpi.com/2073-4360/10/11/1246/s1.
