# Supplementary material for: Bubble Melt Electrospinning for Production of Polymer Microfibers
Source: Polymers (Basel). 2018 Nov 10;10(11):1246. doi: 10.3390/polym10111246 (PMC6401807; doi:10.3390/polym10111246)
Supplement: Supplementary file 1 [file polymers-10-01246-s001.zip › Supporting Information.pdf]

Supporting Information

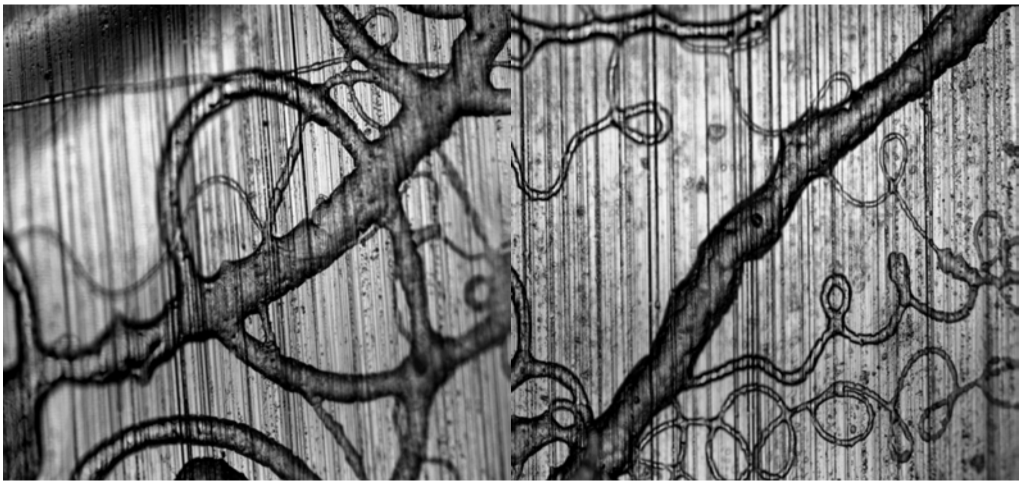

Fig. S1 Optical microscope images of PCL fibers via bubble melt e-spinning.

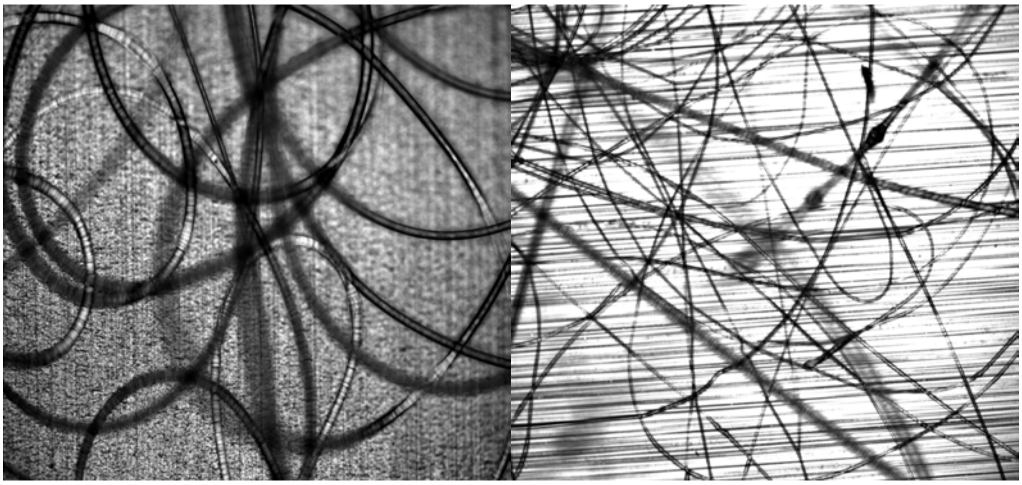

Fig. S2 Optical microscope images of PLA fibers via bubble melt e-spinning.

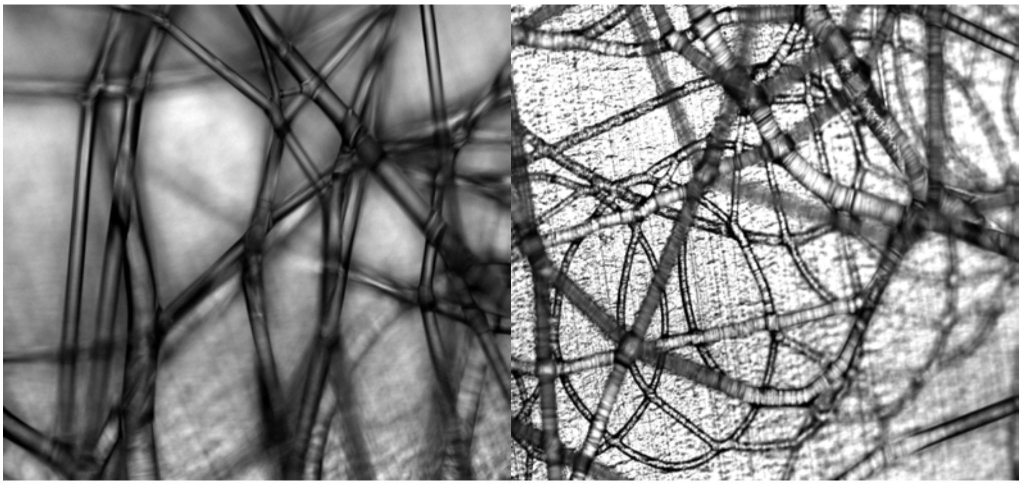

Fig. S3 Optical microscope images of PU fibers via bubble melt e-spinning.
